# Binding Leverage as a Molecular Basis for Allosteric Regulation

**DOI:** 10.1371/journal.pcbi.1002148

**Published:** 2011-09-15

**Authors:** Simon Mitternacht, Igor N. Berezovsky

**Affiliations:** 1 Computational Biology Unit/UNI Research, University of Bergen, Bergen, Norway; 2 Department of Informatics, University of Bergen, Bergen, Norway; Max-Planck-Institut für Informatik, Germany

## Abstract

Allosteric regulation involves conformational transitions or fluctuations between a few closely related states, caused by the binding of effector molecules. We introduce a quantity called binding leverage that measures the ability of a binding site to couple to the intrinsic motions of a protein. We use Monte Carlo simulations to generate potential binding sites and either normal modes or pairs of crystal structures to describe relevant motions. We analyze single catalytic domains and multimeric allosteric enzymes with complex regulation. For the majority of the analyzed proteins, we find that both catalytic and allosteric sites have high binding leverage. Furthermore, our analysis of the catabolite activator protein, which is allosteric without conformational change, shows that its regulation involves other types of motion than those modulated at sites with high binding leverage. Our results point to the importance of incorporating dynamic information when predicting functional sites. Because it is possible to calculate binding leverage from a single crystal structure it can be used for characterizing proteins of unknown function and predicting latent allosteric sites in any protein, with implications for drug design.

## Author Summary

Allosteric protein regulation is the mechanism by which binding of a molecule to one site in a protein affects the activity at another site. Although the two classical phenomenological models, Monod-Wyman-Changeux (MWC) and Koshland-Némethy-Filmer (KNF), span from the case of hemoglobin to membrane receptors, they do not describe the intramolecular interactions involved. The coupling between two allosterically connected sites commonly takes place through coherent collective motion involving the whole protein. We therefore introduce a quantity called binding leverage to measure the strength of the coupling between particular binding sites and such motions. We show that high binding leverage is a characteristic of both allosteric sites and catalytic sites, emphasizing that both enzymatic function and allosteric regulation require a coupling between ligand binding and protein dynamics. We also consider the first known case of purely entropic allostery, where ligand binding only affects the amplitudes of fluctuations. We find that the binding site in this protein does not primarily connect to collective motions – instead the modulation of fluctuations is controlled from a deeply buried and highly connected site. Finally, sites with high binding leverage but no known biological function could be latent allosteric sites, and thus drug targets.

## Introduction

Protein function depends on the balance between different conformational states. This balance can be shifted by many external factors that regulate protein activity, including localized perturbations such as ligand binding or phosphorylation. When the perturbation site is not directly adjacent to the site of altered activity the regulation is called allosteric. A classic example of allosteric regulation is the cooperative ligand binding of many oligomeric proteins, where binding of substrate to one subunit affects the ligand affinity in other identical subunits. The early phenomenological MWC (Monod-Wyman-Changeux) [1] and KNF (Koshland-Némethy-Filmer) [2] models were devised to explain this cooperativity; the first model states that binding stabilizes one of several available states with emphasis on symmetry conservation [3], whereas the latter assumes an induced-fit scenario. Weber showed that both models can be integrated in a general physical framework [4]. Free energy landscape-based descriptions of allostery have introduced the related terms population shift and conformational selection [5], [6]. In a recent review Cui and Karplus gave a clear discussion of the relation between the classical models [1], [2] and the “new views” of allostery [5], [6], pointing out that the MWC/Weber formalism already includes the idea of population shift [7].

The microscopic mechanisms involved in allostery have been studied at different levels of coarse-graining. Analysis of the effect of different types of perturbations has shown some promise in identifying allosteric sites [8], [9]. Ferreiro et al. showed that frustration localized to a few residues facilitates transitions between alternative conformations [10]. Normal modes have been used to quantitatively analyze different energetic and entropic contributions to allostery [11], [12], [13], and also the major components of conformational change [14], [15]. The interaction networks used in normal mode analysis define subunits of coherent dynamics and can be used to identify key residues that maintain this coherence [16], [17]. The network description has also been extended to study transmission of allosteric signals throughout the protein [16], [18], [19], [20], [21]. Caution must however be taken against overly mechanistic interpretations of the networks: allosteric regulation is primarily a thermodynamic process.

An integral part of the modern understanding of allostery is that the states subject to regulation are part of the intrinsic protein dynamics [3], [6], [22], [23], which to some extent is a truism since states not sampled by the native protein would require infinite binding energies to be given finite Boltzmann weights upon binding. A reasonable interpretation of this concept is however that regulation does not require crossing of large barriers: the relevant states are easily reached from the native basin and are occasionally visited also in the absence of effectors. For example, the allosteric conformational transitions are often well described by low frequency normal modes [14], [15]. The existence of purely entropic allosteric proteins [24], where regulation only alters the magnitude of fluctuations around the native state, also shows the importance of intrinsic dynamics. Studies of artificial allosteric inhibitors show that allosteric proteins are often amenable to additional regulation, and that artificial inhibitors stabilize a “naturally occurring conformation” [25]. These observations give hope for identifying allosteric sites based on intrinsic protein dynamics without doing full scale simulations: it seems that knowledge of basic degrees of freedom, such as low frequency normal modes, or some alternative conformations from different crystal structures, gives useful information for finding plausible mechanisms for allosteric regulation.

Our goal is to build a general molecular description of allosteric regulation that allows prediction of biological and latent allosteric sites, as well as catalytic sites, from crystal structures. We restrict our analysis to enzymes regulated by ligand binding, not considering allostery due to metal binding, covalent modifications or interactions with other macromolecules [26]. Ligand-induced allostery can either be heterotropic – when the effector ligand is not the same as the substrate – or homotropic, where the effector is the substrate itself and its binding changes the substrate affinity in other identical subunits of the protein. We include cases where binding of one substrate affects the affinity for a second substrate at the same site, if this change in affinity is associated with a significant change in the protein's structure or dynamics.

To predict allosteric sites we will analyze the ability of different binding sites to couple to the intrinsic dynamics of a protein. Ideally, one would study the thermodynamics of the protein by simulations sampling the relevant parts of conformational space, but limitations in the currently available energy functions, and also in computational power, make this unfeasible. The motions are instead approximated by either normal modes or comparisons between crystal structures representing states with different activity. A large body of research has shown that the functional motions of many proteins are well described by low-frequency normal modes (see the reviews by Ma [14] and Bahar [15]). This does not mean that proteins undergo large-scale harmonic motion, but it shows that low frequency normal modes capture the most important conformational degrees of freedom around the native state – by definition, transitions between distinct free energy minima require barrier crossing [27]. To find potential binding sites we will employ a minimalistic docking procedure to probe the surface of a protein and generate a list of possible binding sites. For each site we estimate the strain on the ligand-protein contacts under the deformations described by low frequency normal modes. The strain is high when the ligand has many contacts with residues that are moving in opposite directions. We say that the site has high *binding leverage* (a mathematical definition is given in the Results section). Ligands binding to such sites have a large potential to affect which states are available to the protein.

In this paper we introduce a fast computational approach that uses binding leverage to predict functional binding sites. The novelty of our method lies in the clear connection between conformational change and binding, and the low computational cost, which allows large-scale analysis. We study two sets of proteins: 15 enzymes with well-documented allosteric regulation, and 226 enzymes from different SCOP domains with annotated catalytic residues. The allosteric enzymes have at least two crystal structures showing the conformational changes involved. We probe the binding pockets of the proteins and find that the biological sites generally have higher binding leverage than other surface pockets. Ligands binding to high leverage sites can couple strongly to conformational changes and are thus able to modulate them. Consequently, we propose that other sites with high binding leverage are either natural or latent allosteric sites. We show that our approach can be applied to comprehensive data sets to detect drug targets in the form of latent allosteric sites. Finally, we analyze a case of purely entropic allostery and find that collective motion probably plays a smaller role here, implying a different mechanism for this type of allostery. Our analysis however indicates that the high buriedness of the effector site is important for this protein.

## Results

### Definition of binding leverage

**Figure 1:**
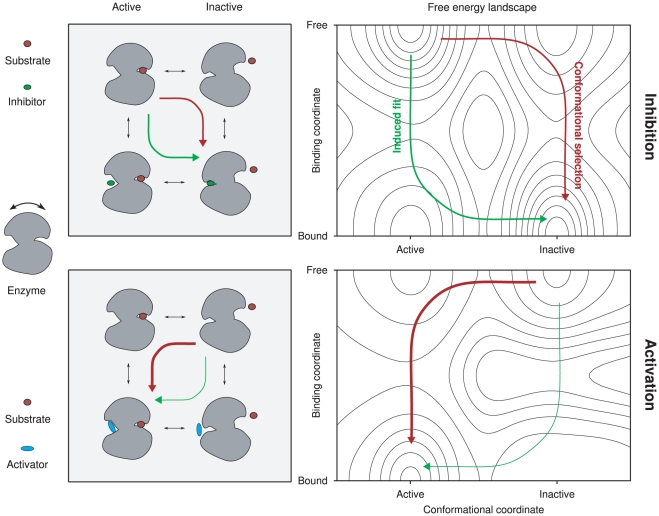
A basic model for allostery. The top left panel shows an enzyme that is allosterically inhibited by ligand binding: the inhibition takes place by stabilization of an inactive conformation. The top right panel shows a putative free energy landscape for this process. The transition from the active to inhibited state can follow one of two main paths, either induced fit (green) or conformational selection (red). The bottom panels show allosteric activation for the same protein. With the geometry of the illustration there will be a large barrier for induced fit, as indicated in the bottom right free energy landscape.

We have illustrated what we see as the most basic mode of allosteric regulation in Figure 1. An enzyme samples a number of conformations, of which some are catalytically competent, while others are not. For illustrative purposes we assume that there is one main active and one main inactive conformation. In the active state the catalytic site has a conformation that allows substrate binding and transformation. In the inactive state the catalytic site is deformed to some extent. Any binding pocket other than the active state that also changes conformation during this transition could potentially be used for allosteric regulation, either activation or inhibition. In our illustration there is one such pocket, which is where the effector binds. Included in the picture is also a putative free energy landscape from which it is clear that there is no intrinsic reason why conformational selection and induced fit should be mutually exclusive mechanisms [28], [29]. In specific cases one could imagine that the barrier to effector binding is very high in one of the states, for example due to steric hindrance, which would then favor one mechanism over the other, but there is no fundamental opposition between the two.

**Figure 2:**
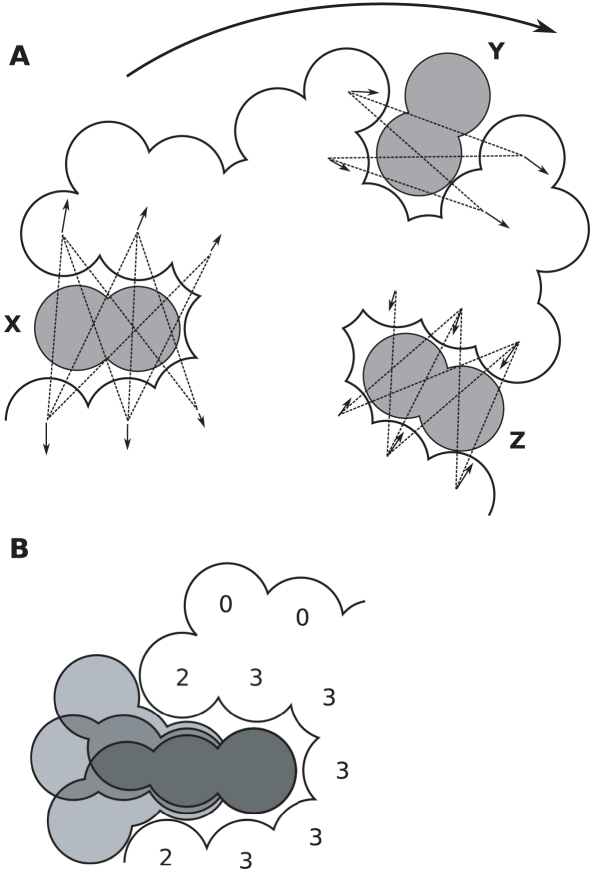
Binding leverage. (A) Illustration of binding leverage for one normal mode. The outline represents the protein surface and the grey dumbbells ligands. The curved arrow shows the general direction of motion in one normal mode and the small arrows the direction of motion for specific Ca atoms in the same mode. Dashed lines indicate pairs of atoms whose interconnecting line crosses the ligand. (B) Illustration of the residue count *f_i_*(*x*) for three probe locations.

To study the ability of a binding site to affect the conformational equilibrium we will introduce a quantity that we call binding leverage. We generate possible ligand conformations by coarse-grained docking simulations where the ligand is a chain of a few C_α_ atoms (see Methods); we call the residues that interact with the ligand in a given conformation a *probe location*. Figure 2A shows part of a protein with three ligands bound, with arrows indicating directions of motion. The motion could correspond to a particular normal mode or be a transition known to be of functional significance. The ligands can affect the local deformation of a site in two ways, either by attracting the surrounding atoms and thus preventing opening or shearing deformations (ligand X), or by sterically blocking the site from closing (ligand Z). Such sites have high binding leverage under the proposed motion. Ligand Y, on the other hand, binds to a pocket that is not deformed, and thus has low binding leverage for the depicted motion. The sites that bind ligands X and Z are allosterically coupled, like the effector and substrate in Figure 1, whereas the binding site for ligand Y can neither function as effector site nor be regulated by the other two sites under these conditions.

To quantify the concept of binding leverage, we use either a vector Δ***x*** describing the difference between two aligned structures, or low frequency normal modes, to represent possible motions (arrows in Figure 2A). Between each pair of C_α_ atoms *i* and *j*, whose connecting line passes within 3.5 Å of any probe atom, we place a spring of length *d_ij_* (dashed lines in the figure). We then measure the change in potential energy of the spring due to motion described by either a normal mode or Δ***x***,
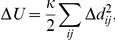
where the indices *i* and *j* run over all relevant atom pairs, and 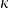 is an arbitrary spring constant. This change in potential energy is meant to represent the cost of deforming the site when the ligand is present and resisting the motion. If Δ*U_k_* represents this change for normal mode *k*, the binding leverage *L_A_* for a set of modes *A* is then calculated as
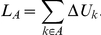
The actual sets *A* used will be described below. The binding leverage calculated using Δ***x*** is denoted *L*_Δ_, and calculated from the one Δ*U* corresponding to that vector.

Binding leverage should not be interpreted literally as an energy, but as an indication of the strength of coupling between ligand binding and functional dynamics. The binding leverage of a site both depends on the range of motion at the site and how tightly bound the ligand is, i.e. how many pairs of residues connect with the ligand. A ligand that binds to a site with high binding leverage has a potential to lock one or more collective degrees of freedom, thus shifting the balance between the states that are sampled along those coordinates. If the difference between these states is of functional relevance, binding to the site can have an effect on activity.

We detect important sites by counting the number of times a residue is part of probe locations with binding leverage above a certain threshold. Figure 2B illustrates how this count works at a site with three probe locations with high binding leverage. The core of the binding site has a higher count than its periphery, which indicates that the core residues are responsible for the high scores of the three probe locations. The count thus gives a rough picture of “hot spots” on the surface of the protein. It does not distinguish between probe sites at the top and bottom of the list – it simply identifies important residues. We call the count *f_i_*(*x*), where the index *i* represents a given residue and *x* is the fraction of ranked probe locations considered. With this definition *f_i_*(1) simply measures the distribution of probe locations, and *f_i_*(0.1) is based on the top 10% probe locations.

We propose the following procedure for finding allosteric sites based on binding leverage (details are provided in Methods): (1) Docking simulations generate a large number of probe locations; similar probe locations are paired to remove redundancy. (2) Relevant motions are calculated, either using normal modes or by comparing crystal structures. (3) The probe locations are ranked by binding leverage or local closeness, and then (4) compared with known biological sites to allow the receiver operating characteristic (ROC) to be measured. (5) Optionally, the measure *f_i_*(*x*) is used to visualize the predictions for different values of *x*.

### Active site prediction

We began our analysis by testing binding leverage on a set of 226 protein domains from different SCOP [30] superfamilies with annotated catalytic sites [31] taken from a paper by Slama et al. [32]. Previous studies have found that catalytic sites are often located between domains of anticorrelated motion [17], which means that they are likely to have high binding leverage. We produced probe locations with the number of simulations set to 10 times the number of residues, and the number of MC steps to 1 000 times the size of the simulation box measured in Å (the box size is set according to the Methods section). The number of atoms in the probe (probe size) was set to 4 universally. These numbers were chosen to make sure that the whole protein surface is sampled and to give a chance for the probe to explore also the deeper pockets.

To rank probe locations we calculated the binding leverage based on the 10 lowest frequency normal modes, *L*_LF10_, and also the local closeness [33] (LC, see Methods). To get a simple statistic of the predictive ability of *L*_LF10_ and LC we measured the area under the ROC curve (AUC) for all proteins, using the ranked probe locations (see Methods). A probe location that involves more than 80% of the catalytic residues of any biological site was considered a positive. For illustrative purposes Figure S1 shows ROC curves for 15 randomly chosen proteins.

For 51 of the 226 proteins there were no probe locations that matched the catalytic site. Using probe size 6 we found the active sites of 10 additional proteins, and with probe size 2 we found 2 more. Manual checks of a few of the remaining 39 proteins showed that these had active sites at dimer interfaces. Such active sites involve residues from more than one chain and are not expected to have detectable binding pockets in single chains.

**Figure 3:**
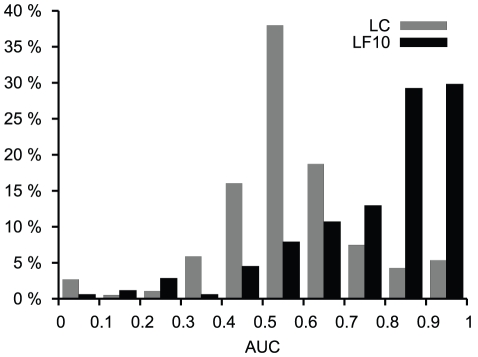
Prediction of catalytic sites. Histograms of AUC values based on catalytic site predictions using LC and *L*_LF10_ for 226 enzymes.

The distribution of AUC values of our predictions – for proteins that did have probe locations matching the target sites – is presented in Figure 3. Out of these proteins 56% had AUC values above 0.8 using *L*_LF10_. The corresponding number for LC was only 10%. These numbers show that knowledge of basic collective motions, described by for example normal modes, greatly helps in predicting catalytic sites.

### Allosteric transitions

We studied 15 allosteric enzymes that were selected using rather strict criteria (see Methods). The proteins chosen are listed in Table 1. We began our analysis of the allosteric proteins by describing the conformational change that could be deduced from crystal structures. By measuring the overlap between the lowest frequency normal modes and the conformational change we found that in some cases up to 90% of the motion is described by only a few normal modes, and in all cases except two it is enough to have 20 modes to describe 50% of the motion (see Figure S2). We determine the collectivity of the conformational transitions using the measure by Brüschweiler, which is 1 for completely collective transitions (rigid body motion) and 1/*N* (where *N* is the number of atoms) for motions involving only one atom [34]. shows that this collectivity is lower for small proteins – where the transition is dominated by tertiary rather than quaternary rearrangements – and for proteins for which the transition is poorly described by low frequency modes. In addition, we analyzed the collectivity of the 10% lowest frequency modes for all 15 proteins (see Figure S3). Except for BGDH and ATCase, the collectivity is on average much higher for the first 10 modes than for the others. As we will show below, the predictions for BGDH using the 10 lowest frequency modes are comparable to those of the other proteins, indicating that binding leverage gives an accurate description even when all of the modes used have low collectivity.

**Table 1:** Simulation parameters and results.

Protein	Chains	PDB entries used	RMSD	Probe size	# sim.	MC steps	Ligands	Probed sites	AUC			
									*L*_Δ_	*L*_FM5_	*L*_LF10_	LC
AdK	1	*1ake, 4ake*	7.1 Å	8	500	100 000	AP5	1/1	0.91	0.91	0.94	0.73
AnthS	2×2	*1i7s, 1i7q*	3.3 Å	4	3 000	300 000	TRP BEZ PYR ILG	7/8	0.97	0.97	0.92	0.75
ATCase	3×2+2×3	1d09, 1rac, *3d7s, 7at1*	6.3 Å	4	12 000	700 000	PAL CTP ATP	15/15	0.68	0.51	0.46	0.24
BGDH	6	*1nr7*, 1nqt, *1hwz*	4.6 Å	6	10 000	300 000	ADP GTP NDP	13/18	0.68	0.73	0.83	0.89
DAHPS	4	*1gg1, 1kfl*	1.5 Å	4	3 000	200 000	PGA PHE	4/8	0.87	0.82	0.79	0.98
DAK	2	*3ju5, 3ju6*	0.7 Å	4	3 000	150 000	ANP ARG	1/2	0.81	0.73	0.85	0.93
G6PD	6	*1cd5, 1hor*, 1hot	4.2 Å	2	4 000	250 000	AGP 16G	12/12	0.69	0.68	0.70	0.70
NADME	4	*1efk, 1gz3*	0.8 Å	2	10 000	600 000	ATP FUM	8/8	0.69	0.74	0.77	0.68
PFK	4	*3pfk*, 4pfk, *6pfk*	1.7 Å	4	4 000	200 000	PGA F6P	8/8	0.80	0.71	0.81	0.65
PGDH	4	*1psd, 1yba*	3.4 Å	4	10 000	400 000	NAD AKG SER	11/12	0.69	0.60	0.62	0.61
PKA	1	*1j3h, 1atp*	2.6 Å	6	1 000	200 000	ATP	1/1	0.98	0.97	0.98	0.95
PTP1B	1	*2hnp*, *1aax*, 1t49	0.8 Å	2	1 000	100 000	BPM 892	3/3	0.77	0.71	0.74	0.71
SSUPRT	4	*1xtt, 1xtu*	1.9 Å	4	4 000	150 000	CTP U5P	8/8	0.94	0.84	0.75	0.73
ThrS	2	*1e5x, 2c2b*, 2c2g	2.1 Å	4	1 500	200 000	SAM LLP	6/6	0.87	0.74	0.79	0.93
TrpS	2×2	*1bks, 3cep*	1.7 Å	2	3 000	300 000	G3H PLP IDM	6/8	0.89	0.92	0.58	0.82
							Average:		0.82	0.79	0.79	0.79

AUC averages were calculated without the outlier ATCase. Ligand names are taken from the PDB files. Italicized PDB codes indicate the pair of structures used to calculate RMSD and difference vector. The column labeled “# sim” indicates the total number of simulations performed. “Probed sites” indicates how many of the binding sites our probe managed to reproduce. The AUC values are averages over two independent runs with different random number seeds. The differences between the total averages (bottom row) of the two runs were in the range 0.005–0.015.

Most of the proteins in Table 1 (ATCase, DAHPS, DAK, BGDH, G6PD, NADME, PFK, PGDH and SS-UPRT) display classical oligomeric allostery. The majority are both homotropically and heterotropically regulated. They are all described well by low frequency normal modes, as can be seen from Figure S2. The heterotetrameric enzymes anthranilate synthase (AS) and tryptophan synthase (TrpS) share the property that they have two different active sites and that activity at one site controls activity at the other. In both cases this allows the product of one reaction to be used efficiently for the second reaction [35], [36], [37], [38]. In addition, AS is inhibited by tryptophan binding to a third site. Low frequency normal modes describe the allosteric transition of both these proteins well.

Two of the analyzed proteins are kinases: adenylate kinase (AdK) and protein kinase A (PKA). Their dynamics have been analyzed extensively in the literature [27], [39], [40], [41], [42], [43], [44], [45], [46]. They are not allosteric proteins in the classical meaning – ATP and substrate bind to the same site – but in both cases binding of ATP and/or substrate causes a large conformational change, and they are therefore often described as allosteric. Previous research has shown that the closing of AdK is well described by a few low-frequency normal modes [47], and we found that this is also the case for PKA (Figure S2).

The enzyme protein tyrosine phosphatase 1B (PTP1B) is inhibited by an artificial effector that immobilizes an active site loop [48]. The localized nature of this motion is reflected in the poor overlap with low frequency modes (Figure S2).

Binding of S-adenosylmethionine (SAM) activates the dimeric enzyme threonine synthase (ThrS) [49]. The main component of the conformational change is asymmetric, only one of the sites is activated upon SAM binding, whereas the other remains largely in the apo conformation [50]. This asymmetry probably explains why the conformational transition is poorly described by low frequency normal modes.

### Overall binding site characteristics

To analyze the binding leverage of the allosteric enzymes we first generated probe locations. Depending on the size of the protein, the number of simulations varied between 500–12 000 to allow the probe to sample all parts of the surface. We ranked probe locations by four different measures, *L*_Δ_, *L*_FM5_, *L*_LF10_ and LC. The three binding leverages *L*_Δ_, *L*_FM5_, and *L*_LF10_ are calculated using the difference vector Δ***x***, the 5 normal modes that overlap the most with functional motion (FM5), and the 10 lowest frequency normal modes (LF10), respectively. For each protein we listed all biological ligands, and defined the binding sites by measuring which residues had at least one atom within 3.5 Å of the ligand (protein and ligand coordinates were taken from a crystal structure with ligand). To measure ROC curves we defined positives as probe locations with more than 40% of the residues of any biological binding site. The AUC value only gives a lower bound to the accuracy, since some of the “false positives” are likely latent sites, which we also want to be able to find. On the other hand, since there is evolutionary pressure to optimize allostery, biological sites should be easier to find than latent ones.

The results of the analysis including the simulation parameters are presented in Table 1. ROC curves for the 15 proteins are shown in Figure S4. On average, the performance is similar between the measures in Table 1, but it can be quite diverse for individual proteins. Surprisingly, the large difference between *L*_LF10_ and LC seen for catalytic site prediction disappeared. We attribute this to the generic definition of binding site used, compared to the well-annotated catalytic residues in the previous set. The fact that *L*_LF10_ is comparable to *L*_Δ_ indicates that regulatory sites can be found from a single structure without experimental knowledge of conformational change.

Initially, we were not able to generate probe locations matching some of the biological sites, usually due to them being too buried or the ligand being large. To reach deep binding pockets we increased the length of the simulations and reduced the probe size (for TrpS for example). Protein structures that have large ligands, such as the bisubstrate analog in the active state crystal structure of AdK (PDB entry 1ake), require larger probe sizes. As far as possible we use apo structures for the docking simulations. In the end we were only unable to match the phenylalanine binding-pocket in DAHPS and some of the NADP and GTP sites in BGDH.

The allosteric transition of the protein PTP1B is not described very well by low frequency normal modes (see Figure S2). In spite of this, the prediction results using *L*_LF10_ and *L*_FM5_ are comparable to those of *L*_Δ_. All three ROC curves (see Figure S4) increase rapidly and then flatten out, because the active site does have high binding leverage while the artificial allosteric site does not. As we showed above, the active sites in single enzyme domains usually have high binding leverage, regardless of if the protein has been shown to be allosteric or not. We attribute the difficulty predicting the allosteric site to a combined effect of a shallow binding pocket and the localized conformational changes involved.

**Figure 4:**
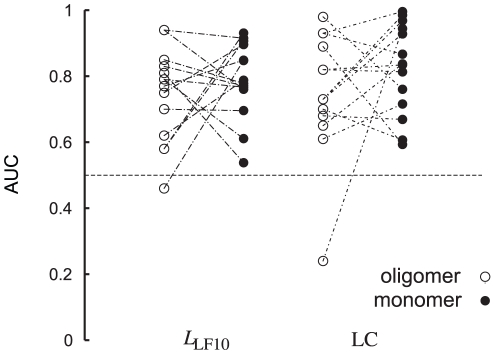
Analysis of single chains. Comparison between AUC values obtained for analyses of oligomeric enzymes and single chains (monomers) taken from the oligomeric structure. ATCase, which has very poor prediction results in the oligomeric analysis, behaves well in the single chain analysis (see also Table 2).

We analyzed single protomers from the oligomeric proteins to see if these could be used to improve predictions for the problematic proteins above. The results are presented in Table 2 and a comparison between the oligomer and monomer analyses in Figure 4. In this situation there was no problem finding all binding pockets in BGDH and DAHPS. Even though we did not take into account that some parts of the monomer are buried in the oligomer, some of the proteins had clear improvements in AUC values in this analysis, in particular ATCase for which performance was poor when analyzing the full protein.

**Table 2:** Analysis of monomers. For each protein 1 000 simulations of 100 000 MC steps each were performed.

Protein	Probe size	Ligands	Probed sites	AUC	
				*L*_LF10_	LC
Anths TrpE	4	BEZ PYR TRP	3/3	0.91	0.83
Anths TrpG	4	ILG	1/1	0.93	0.81
ATCase	4	PAL	1/1	0.92	0.97
BGDH	4	ADP GTP NDP	3/3	0.78	0.87
DAHPS	4	PGA PHE	2/2	0.76	0.93
DAK	4	ANP ARG	2/2	0.85	0.94
G6PD	2	AGP 16G	2/2	0.61	0.76
NADME	2	ATP FUM	2/2	0.70	0.61
PFK	4	PGA F6P	2/2	0.77	0.59
PGDH	4	AKG NAD SER	3/3	0.85	0.67
SSUPRT	4	CTP UP5	2/2	0.54	0.84
ThrS	4	SAM LLP	2/3	0.79	0.72
TrpS α	2	IDM	1/1	0.90	0.99
TrpS β	2	SRI IDM PLP	3/3	0.77	0.996

Before proceeding to specific examples we note that the protein ATCase stood out among the 15 proteins in Table 1; here our predictions were considerably worse than random for some of the measures. With 12 chains, this was the largest protein in our set. The ATP/CTP regulatory site is peripheral, and ranked very low with all our measures. The top ranking sites in our analysis are at chain interfaces that undergo large rearrangements in the allosteric transition. It seems reasonable that ligands that bind to these crevices could stabilize either active or inhibited conformations. When we analyzed one of the catalytic subunits by itself the AUC value for *L*_LF10_ increased from 0.46 to 0.92.

### Analysis of specific allosteric proteins

The above analysis was somewhat abstract in that we assigned a single scalar to evaluate the predictive abilities of our measures, and ultimately the correctness of our model of allostery. To get a better picture of how our measures work we performed a detailed analysis for a few of the proteins (PKA, ThrS, BGDH, PFK). To aid in the analysis we will show *f_i_*(*x*) for the different proteins. The value of *x* will be chosen such that all biological sites are covered.

**Figure 5:**
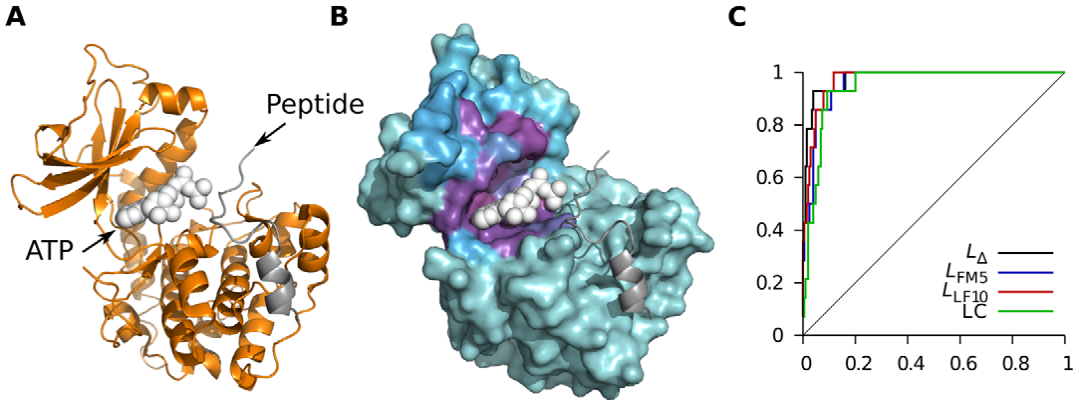
Analysis of protein kinase A (PKA) using *L*_LF10_. (A) Cartoon of protein structure. Protein coordinates are taken from PDB entry 1j3h and ligand coordinates from 1atp. (B) Residues colored by *f_i_*(0.04), with cyan representing *f_i_* = 0 and magenta the highest recorded *f_i_*. (C) ROC curves obtained with the four measures in Table 1.

PKA undergoes a relatively large conformational change upon substrate binding, as is common for many kinases [40]. Figure 5A shows the structure of PKA together with its two substrates. It has a kidney shape with one smaller and one bigger lobe that close over the active site when both ATP and protein substrate are present. Binding of either substrate causes partial closing of the active site [40] and experiments have shown that binding of one substrate increases affinity for the other [41]. Figure 5B shows that the active site has the highest binding leverage, in line with the fact that binding here causes large conformational change. The whole peptide binding site is not captured but the “hot spot” covers part of it. Since the peptide is relatively large we do not expect its entire binding site to have high binding leverage, and it was not included in the ROC analysis in Table 1. For reference, the corresponding ROC curve is shown in Figure 5C. To assess the significance of the particular choice of crystal structure used we also analyzed PDB entry 1atp. Probes of size 6 were not able to find the active site in this closed structure, but probes of size 2 did. The AUC values were 0.90, 0.80, 0.84, and 0.99 for *L*_Δ_, *L*_FM5_, *L*_LF10_, and LC respectively, which is a decrease for all measures except LC. This decrease is probably primarily due to the difficulty of docking using a closed structure. The normal modes of a closed structure might also be less useful [47].

**Figure 6:**
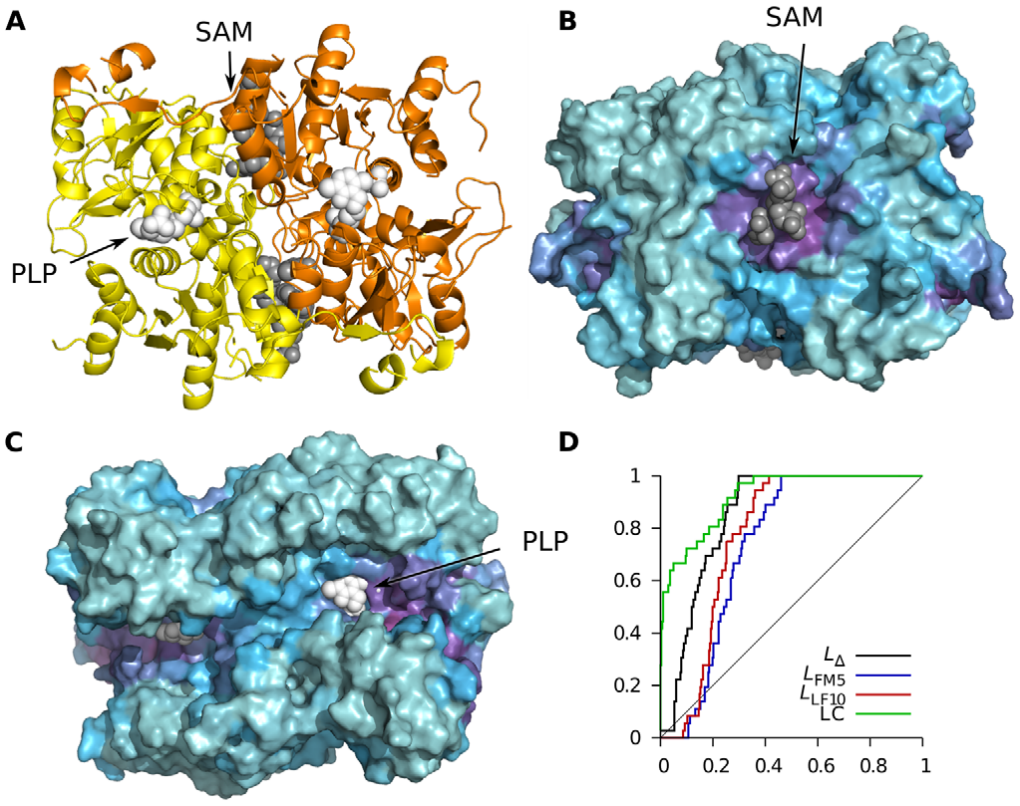
Analysis of threonine synthase (ThrS) using *L*_LF10_. (A) Cartoon of structure with the coenzyme PLP drawn with white spheres and the SAM molecules with grey spheres. Protein coordinates are taken from PDB entry 1e5x and ligand coordinates from 2c2b. (B) Rotated view showing the SAM binding site, colored according to *f_i_*(0.42). (C) Same view as (A) showing *f_i_*(0.42) for active site cleft. (D) ROC curves.

ThrS synthesizes threonine from O-phosphohomoserine with pyridoxal phosphate (PLP) as coenzyme. As mentioned, it is allosterically activated by S-adenosylmethionine (SAM) binding, which causes conformational change at the PLP site [49], including the PLP molecule itself [50]. In total four SAM molecules bind to the dimer, two per protomer, at the interface between the two chains (Figure 6A). The active site is in the large cleft dominating one face of the structure. As measured by *f_i_*(0.34) (Figure 6B & C) the SAM binding site is well defined and relatively isolated, but the PLP site is part of a larger region with high binding leverage. It is possible that binding anywhere in this cleft could affect the dynamics and activity of the protein. The wide spread of sites with high binding leverage around the active site explains the poor AUC values for this protein in Table 1, also illustrated by the ROC curve in Figure 6D. We note that even though the allosteric transition was not particularly well described by low frequency normal modes (Figure S2), *L*_LF10_ captures the important regions of the protein. LC does however do a better job than binding leverage at pinpointing the exact location of the important sites.

**Figure 7:**
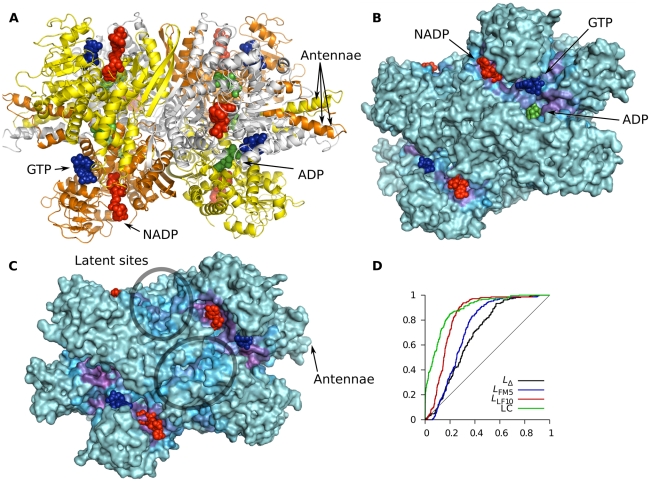
Analysis of bovine glutamate dehydrogenase (BGDH) using *L*_LF10_. (A) The hexameric structure of BDGH with ligand atoms drawn as spheres. Red ligands are NADP, blue GTP and green ADP. The individual chains are colored yellow, orange and white within each trimer. 1nr7 was used for protein coordinates, and NADP, GTP and ADP were taken from PDB entries 1hwz and 1nqt. The antenna helices of three of the chains are indicated with arrows. (B) Slightly rotated view showing the active and both allosteric sites, colored according to *f*_i_(0.25). (C) When more probe locations are included in the analysis, latent sites emerge, as exemplified here by *f*_i_(0.41). (D) ROC curves.

The allosteric regulation of bovine glutamate dehydrogenase (BGDH) is complex and involves both homotropic and heterotropic effects (Figure 7A). This hexameric protein is negatively cooperative with respect to coenzyme binding (NADP+), and is primarily regulated by GTP (inhibition) and ADP (activation). Additional allosteric ligands have also been identified [51]. Each chain consists of three subdomains: the Glu, NAD and antenna domains. Rotation of the NAD domain towards the Glu domain closes the catalytic cleft that lies between them. The cleft needs to close to initiate catalysis and then open to release the reaction products [52]. The opening of the cleft varies quite significantly throughout the asymmetric unit of the apo crystal, showing that the protein can visit a variety of states in its unliganded form [53]. ADP binding is compatible with both closed and open structures [54], and is believed to facilitate the transition between the two [53]. GTP only binds to a closed conformation and probably locks the enzyme in this state [54]. In addition to the ones discussed here, there are several allosteric effectors whose binding sites are unknown [51]. There are also artificial inhibitors that bind to the central core of the hexamer [55].

The ROC curves in Figure 7D show that robust predictions can be made for this protein using both *L*_LF10_ and LC, based on the sites that were matched by probe locations. Figure 7B shows *f*_i_(0.25) for BGDH (based on *L*_LF10_ ranks), demonstrating that both the active site and the cleft surrounding the ADP site have high binding leverage. GTP binds in the cleft connecting the two sites which is also detected by *f*_i_(0.25). Even though our probe was not able to match all binding sites we see here that all the known biological sites have high *L*_LF10_. By increasing the number of probe locations taken into consideration we found several possible latent allosteric sites: In Figure 7C we see that *f_i_*(0.41) gives two additional sites with threefold symmetry at the waist of the protein, i.e. the interface between the two trimers. As mentioned, artificial allosteric effectors have been found to bind at the center of the protein [55], which does not seem to be accessible to our probe, but the waist sites we found are in the same general area. This extended analysis also points out the entire bases of the antenna helices to be important (partly shown in Figure 7C). These helices are believed to be involved in the negative cooperativity of NAD binding and also in making catalytic turnover more efficient [53].

**Figure 8:**
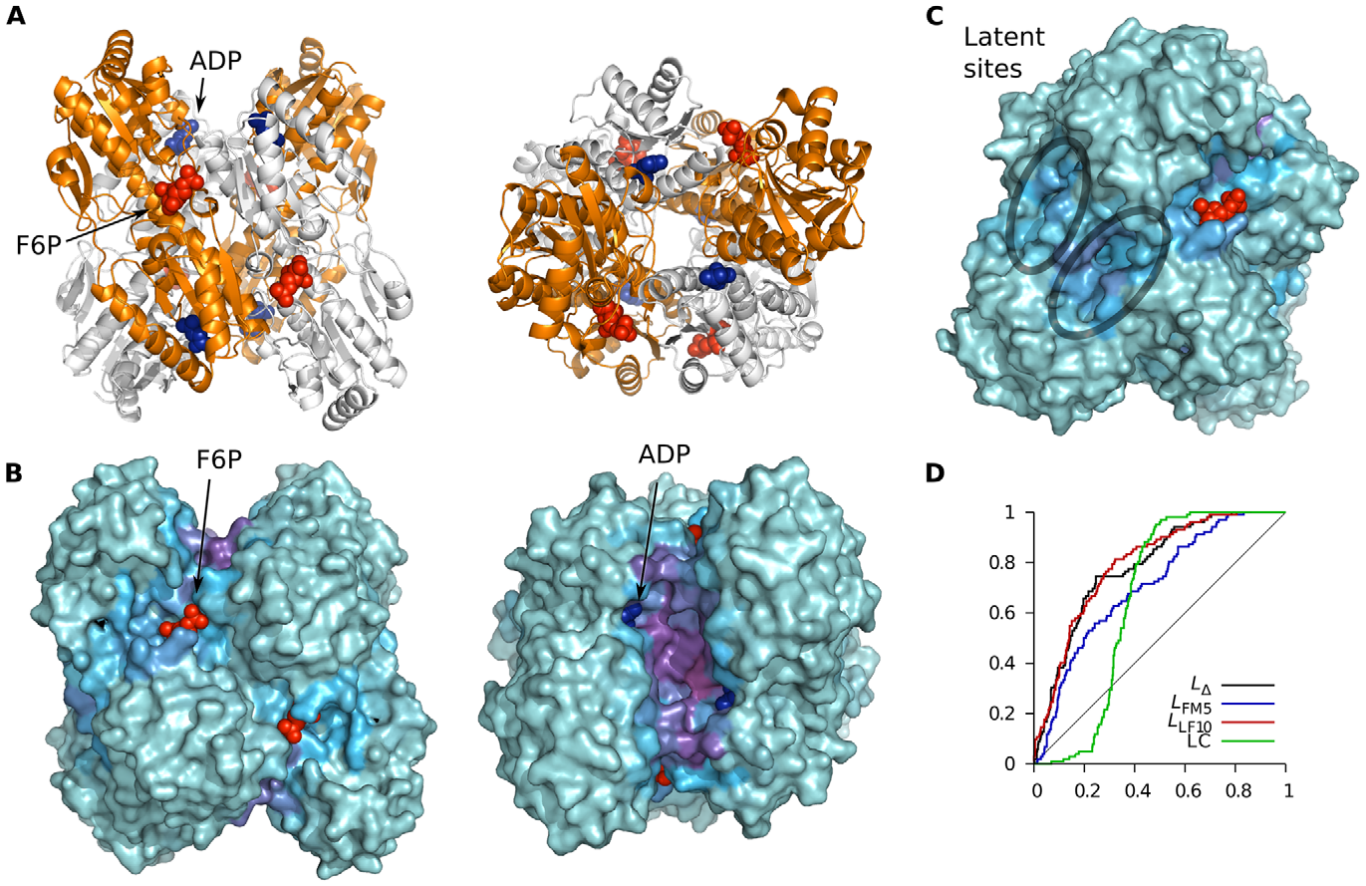
Analysis of phosphofructokinase (PFK) using *L*_LF10_. (A) Two views of the tetrameric structure. Blue spheres represent the activator ADP and red spheres the substrate F6P. Protein coordinates were taken from PDB entry 3pfk, coordinates for F6P from 4pfk, and for PGA from 6pfk. (B) Same views as in (A) but now showing the surface colored by *f_i_*(0.22). (C) Latent sites can be seen in a slightly rotated version of the left half of (B). (D) ROC curves.

The tetrameric enzyme phosphofructokinase (PFK) is allosterically inhibited by phosphoenolpyruvate (PEP) and activated by ADP binding to the same site [56]. It is cooperative with respect to binding of the two substrates, fructose-6-phosphate (F6P) in the presence of PEP [57]. The crystal structure with the allosteric activator ADP present (4pfk) is in principal identical to the apo structure (3pfk) [58], indicating that the activator simply stabilizes the active state. An inhibited structure has also been determined by crystallizing the protein with a PEP-analog (6pfk). The binding of inhibitor primarily causes a quaternary structural change – two dimers rotate rigidly with respect to each other [56]. Within the subunits, two helices in the effector-binding domain shift positions, otherwise the chains are largely rigid. The active site lies at the dimer-dimer interface and is thus affected by the quaternary change (see Figure 8A). Our analysis (Figure 8B) showed that the active site F6P ligand is at the center of a “hot spot” and the regulatory PEP/ADP sites are at the periphery of the central “pore” that has high binding leverage. In addition to the biological sites we detected a possible latent site with fourfold symmetry (Figure 8C), which is located at a chain-chain interface.

### Allostery without conformational change

**Figure 9:**
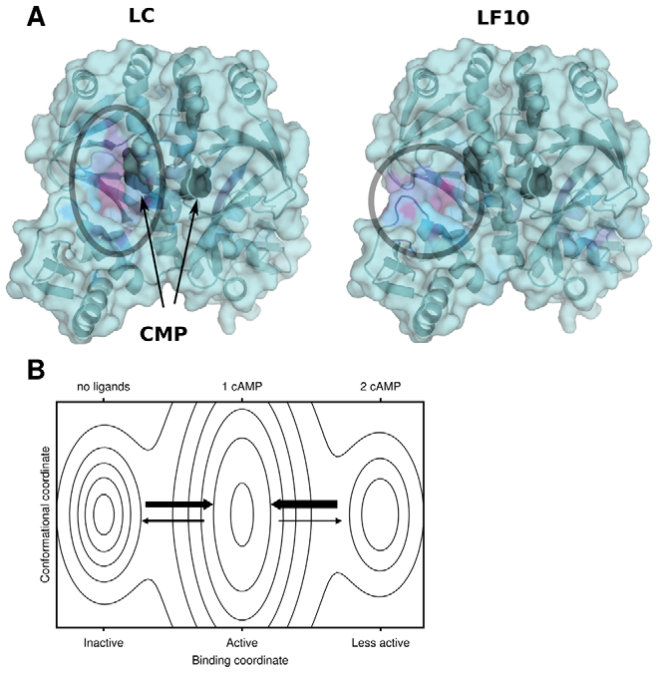
Catabolite activator protein (CAP). (A) Binding analysis of CAP based on PDB entry 1g6n. The coloring is based on *f_i_*(0.1). To help the eye the most important sites have been marked with circles. The dimer is symmetric; the sites hidden in this view have similar properties to the ones shown. The location of the cAMP ligand in the crystal structure is marked by black spheres. (B) Putative free energy landscape at an intermediate cAMP concentration. The *x*-axis indicates the number of cAMP molecules bound, and the *y*-axis conformational degrees of freedom. We indicate that the conformational entropy is highest when one cAMP is bound by the wider minimum.

Lastly we turn to the dimeric catabolite activator protein (CAP) – a transcriptional activator allosterically regulated by cAMP-binding [59]. It is negatively cooperative with respect to cAMP binding, but displays no significant conformational change upon binding. NMR experiments have however shown that binding of one cAMP molecule increases fluctuations, i.e. entropy, and that binding of a second cAMP molecule quenches these fluctuations [24], and thus the affinity is lower for the second molecule. We have illustrated the free energy landscape at an intermediate ligand concentration in Figure 9B. The distinction between allosteric pathways illustrated in Figure 1 does not apply here.

We did 1 500 probe simulations of 100 000 MC steps, using a probe of size 2 and protein coordinates from PDB entry 1g6n. We ranked probe locations using LC and *L*_LF10_. AUC values are 0.61 for *L*_LF10_ and 0.98 for LC. Figure 9A shows *f_i_*(0.1) for both measures. The cAMP ligand is marked in the figure by black spheres. It is clear both from the figure and the AUC values that LC predicted the allosteric sites well, whereas *L*_LF10_ did not. The top site predicted by binding leverage is at the interface between two subdomains. As far as it is known this cleft has no binding partner. The actual cAMP binding site is more deeply buried and not directly connected to collective motion, which could mean that the allostery of this protein primarily involves other types of motion. Experiments show that both slow (0.1–1 s) and fast backbone fluctuations (1 ps–1 ns) contribute to the negative cooperativity of cAMP binding, and it seems that the quenching upon binding of the second cAMP ligand is the primary component of negative cooperativity [24]. The measure LC can be used to locate deeply buried, highly connected sites, which are presumably well suited for inducing the type of global rigidification observed for this protein.

## Discussion

It is well understood that catalysis and allosteric regulation takes advantage of the motions inherent to the native protein (by motions we mean both pure fluctuations and visits to nearby local free energy minima). Ligands binding at catalytic and allosteric sites should be able to couple to these motions. Until now, this understanding has however not been used to predict functional sites, the analysis has instead focused on characterizing the properties of individual proteins. We introduce the measure binding leverage to quantify the coupling between a ligand and conformational transitions of the protein. We use MC simulations to generate probe locations on the surface of the protein, and then measure the binding leverage for these using either low frequency normal modes or motions derived from paired crystal structures. We analyzed a set of 226 diverse well-annotated catalytic domains, and found that binding leverage gives much better predictions of catalytic sites than the static measure local closeness, which we introduced in an earlier study [33]. The analysis of allosteric proteins showed that the binding leverage approach predicts most of the allosteric binding sites studied. We illustrated by some specific examples that our statistical analysis underestimates the quality of the predictions, both because the “false positives” can be understood as possible latent allosteric sites, and because the hot spots match the biological binding sites – also those that were not discovered by comparing the residues in the probe locations with the biological sites. The ability to locate latent allosteric sites is useful for drug design.

The fact that two different ligands can have opposite allosteric effects at the same site, as for PFK, shows that some sites are intrinsically better coupled to the dynamics of the protein and depending on ligand the effect can go either way (see also Figure 1). Furthermore, two of the four proteins we analyzed in detail (BGDH and PFK) are clear examples of cases where at least one of the modes of regulation simply involves stabilizing a conformation also found in a crystal structure without the effector. Binding leverage was devised specifically to detect sites with such abilities.

We demonstrated that it is sometimes easier to predict important sites by looking at single chains rather than full oligomers. The ancestors of oligomeric enzymes were most likely monomeric enzymes of related function. A common path for evolving allostery is probably first formation of oligomers to allow homotropic cooperativity and then the addition of heterotropic regulation, which can take advantage of the collective motions already involved in homotropic regulation. Thus, the catalytic site is often the only site of significance in a monomer and will be easier to identify when the monomer is isolated – the allosteric sites however sometimes only make sense in the context of the oligomer. We recommend a combination of both approaches when looking for catalytic and heterotropic allosteric sites for uncharacterized oligomeric proteins.

We also analyzed the transcription factor CAP, which displays “purely entropic” allosteric regulation. For this protein we found that binding leverage does not predict the allosteric site very well, whereas the purely geometrical measure local closeness does. Even though it is hard to draw any definite conclusions from a single example, a plausible explanation in this case is that allostery without observable conformational change does not involve the collective motion associated with the canonical examples. Since local closeness finds deep surface pockets with high residue inter-connectivity, it might be a better measure than binding leverage for detecting sites from which alterations of local fluctuations can propagate through the protein. When available, other proteins with entropically driven allostery should be analyzed with respect to local closeness to verify whether this conclusion is valid. There is of course always an entropic component involved in allosteric transitions. Hypothetically, the cases in Table 1 where binding leverage gives poorer predictions than local closeness might have a larger entropic component than the cases where binding leverage works well.

As pointed out in the introduction, intraprotein signaling network descriptions do not capture the thermodynamic nature of allosteric regulation. Allosteric regulation depends on conformational transitions between closely related states: knowing the motions involved is often enough to understand how effector ligands regulate activity. We have shown that, for many proteins, one can make reliable predictions of different types of binding sites with a method that only relies on finding binding pockets and measuring how well these pockets are connected to the collective dynamics of the protein. To our knowledge this is the first time the connection between dynamics and functional sites has been analyzed at such a large scale. Prediction of allosteric and functional sites is important for characterizing proteins of unknown function and for locating potentially druggable sites.

## Methods

### Local closeness

In a previous paper we defined the centrality measure local closeness (LC) to predict binding site residues from a residue interaction graph [33]. LC is a minimalistic purely geometric measure that can find catalytic and allosteric sites in a large range of proteins. In the residue interaction graph each node represents a residue and edges represent interacting residues. For a given node, let *n_k_* denote the number of nodes whose shortest distance from the node is exactly *k*. The local closeness for the node is then defined as *C* = *n*_1_+*n*_2_/4+*n*_3_/9+*n*_4_/16 (see the original paper for details [33]).

### Surface probe simulations

We begin the analysis of a protein by probing its surface for potential binding sites using coarse-grained docking simulations. Both protein and ligand are represented as C_α_ backbones. We call the ligand a *probe*, and the number of atoms in the probe the *probe size*. The probe moves freely in the simulations, but for computational efficiency the protein conformation is held completely fixed. The distance between sequential probe C_α_ atoms is kept fixed at 3.8 Å and bond angles in the range 90° to 180° are allowed. The C_α_-C_α_ interaction has the form of a square well attractive potential, for each pair of atoms, in the range 5.5 to 8 Å with depth *ε* = 0.75 *k*_B_ *T*. To decrease the risk of the probe being sterically trapped in deep pockets, the repulsion between atoms is increased in steps. The repulsive energy is +3*ε* for atom-atom distances between 5 and 5.5 Å and +10*ε* between 4.5 and 5 Å. Distances shorter than 4.5 Å are not allowed. The boundary conditions are periodic and the size of the cubic simulation box is set to twice the maximum size of the protein along any of the *x*, *y* or *z*-axes.

We run short MC simulations of this model, with ∼100 000 MC steps, using the Metropolis algorithm [60]. The MC updates for the probe include rigid body rotations and translations. Bond angle updates are used for probe sizes ≥3, and torsion angle updates for probe sizes ≥4. The simulations are started from random configurations with no contacts between probe and protein. The end configuration of each simulation is used to define a binding site candidate. All protein C_α_ atoms interacting with the probe in this configuration define a list of residues, which we call the *probe location*. Because of the simplicity of the model the simulations are fast – on a modern desktop PC (as of 2010) the time needed to generate one probe site is of the order of 1 s (including disk I/O, etc). For large proteins the number of simulations needed to sample the whole protein surface is larger than for small proteins, and more MC steps are needed to traverse a large simulation box. Longer simulations can also be necessary to allow sampling of deeply buried pockets. Probe sizes in the range 2–8 are used. This should correspond to typical ligand sizes up to small peptides and dinucleotides. Using a somewhat oversized probe size increases the chance of covering the relevant residues at a given site, but steric hindrance makes it more difficult to reach deep binding pockets.

A set of docking simulations generally produces some redundant probe locations. To refine the list of locations we score the probe locations by LC: we first calculate LC for all residues in our protein. For each probe location we then identify the 10 residues with highest LC and score the site by the average LC of those residues. We compare two probe locations, *A* and *B*, using the Jaccard similarity 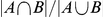. The similarity is 1 when *A* and *B* are identical and 0 when they contain no common elements. Before any analysis is done we merge locations that have a Jaccard similarity higher than 0.7 keeping the location with the higher local closeness score and merging the most similar ones first.

### Normal mode analysis

The second step in the analysis is to find relevant motions for a given protein. We use either a comparison between crystal structures or normal modes. The normal mode analysis is done using C_α_ elastic networks in the Molecular Modeling Toolkit (MMTK) [61]. We calculate vibrational modes using the default parameters of MMTK. In all analyses we discard the 6 trivial zero frequency modes. We denote the *j*:th normalized normal mode ***e*** *_j_* and the normalized difference vector between two aligned crystal structures 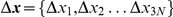. The modes are sorted by frequency.

To analyze to which extent an allosteric transition is described by low frequency normal modes we use two related measures [62]. First we measure the overlaps *I_j_* = Δ***x***⋅***e*** *_j_*/|Δ***x***| |***e*** *_j_*| between the individual normal modes and the conformational transition described by the crystal structures. The overlap is 1 when the mode under consideration describes the whole transition, and 0 when the mode is orthogonal to the transition. The sum of all squared overlaps is 1 since the normal modes form a complete basis set. By checking how fast this sum (the cumulative overlap) approaches one, one can assess to what extent the lowest frequency normal modes describe the transition.

### Statistical analysis

The third step in our analysis is to score the probe locations by binding leverage or LC and the fourth and final step is a statistical analysis of the probe locations, done by comparing with known biological binding sites. A probe location that scores above a certain threshold is considered a potential binding site. We define probe locations that contain a certain number of residues from any biological site as positives and then calculate the receiver-operating characteristic (ROC), which measures the true positive rate versus the false positive rate as the threshold is varied. The correctly classified binding sites at a given threshold are true positives. The true positive rate is the number of true positives out of the positives, and the false positive rate is the number of false positives divided by the number of negatives. The area under the ROC curve (AUC), when the false positive rate is varied from 0 to 1, is 1 for a perfect predictor and 0.5 for a random one.

### Selection of allosteric enzymes

We only analyze *enzymes* that are regulated by *ligand binding*. There has to be at least two structures available in the PDB, one regulated (either activated or inhibited) and one that is not regulated. The active and allosteric sites have to be unliganded in at least one of the structures, respectively, although in some cases cofactors are present in all available structures. Some of the proteins were taken from the allosteric benchmark set [63], and others were found by literature searches. The reason we left out most of the allosteric benchmark set is because only about one third of the proteins are enzymes; we exclude the different classes of signaling proteins where allosteric regulation changes the interaction with macromolecules like DNA, RNA and other proteins. Furthermore, some of the enzymes in that set are regulated by phosphorylation or didn't have apo structures available. We also skipped proteins where the regulation caused a shift in oligomerization state.

## Supporting Information

Figure S1 **ROC curves for 15 randomly chosen proteins out of the 226 in the set by Slama et al.** [**32**] **.**
(EPS)

Figure S2
**Overlap with normal modes.** Cumulative overlap of the 10% lowest frequency normal modes with the difference vector between crystal structures. The pairs of PDB entries used to describe the motion are given in the figure. Each protein has two curves, calculated using the normal modes of either structure. Brüschweiler's collectivity measure 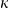 of the transition between two crystal structures is printed in the figure [34].(EPS)

Figure S3 **The collectivity (by Brüschweiler's measure) of the 10% lowest frequency normal modes for all the analyzed allosteric proteins.** The protein BGDH and ATCase are marked because they have relatively low collectivity for their first 10 modes. These are also the two largest proteins in the set.
(EPS)

Figure S4 **ROC curves for the 15 allosteric proteins, measured for the three leverages (** ***L*** **_Δ_,** ***L*** **_FM5_ and** ***L*** **_LF10_) and local closeness (LC).**
(EPS)
